# Iodine solubility and speciation in glasses

**DOI:** 10.1038/s41598-019-44274-4

**Published:** 2019-05-23

**Authors:** M. R. Cicconi, E. Pili, L. Grousset, P. Florian, J. C Bouillard, D. Vantelon, D. R. Neuville

**Affiliations:** 1 Institut de Physique du Globe de Paris, Équipe Géomatériaux, CNRS-UMR7154, Sorbonne Paris Cité, 1 rue Jussieu, 75005 Paris, France; 2 CEA, DAM, DIF, F-91297 Arpajon, France; 3 CNRS, CEMHTI UPR3079, Université d’Orléans, F-45071 Orléans, France; 4 IMPMC - Sorbonne Université, 4 place Jussieu, 75252 Paris, cedex 05 France; 5 SOLEIL Synchrotron, L’orme des merisiers, Saint Aubin BP48, 91192 Gif-sur-Yvette, Cedex France

**Keywords:** Geochemistry, Structure of solids and liquids

## Abstract

The study of iodine in glasses and melts is critical in many areas, from geosciences to materials science to waste management. Glasses in the ternary system Na_2_O-B_2_O_3_-SiO_2_ were studied with the goal of identifying a glass matrix able to dissolve large quantities of this element, and to identify the main parameters affecting the solubility of iodine. Two sets of experiments were carried out: the first one with the aim of determining the solubility limit of iodine, and the second one to identify the structural variations occurring within the glass network upon iodine incorporation, and to identify the parameters influencing the most both iodine solubility and speciation. We demonstrated that there is a strong dependence of iodine incorporation on bulk chemistry and glass physical properties. A solubility limit of ~5 mol% I has been assessed for B_2_O_3_-rich glasses and of ~1 mol% for SiO_2_-rich ones, and this composition dependence has been explained by considering the fragility parameter of the glass network. Structural variations in the iodine local environment and in the glass network were characterized by Raman, X-ray Absorption Spectroscopy, and ^11^B NMR. Spectroscopy data point out the coexistence of different I species within the glasses, with iodide being the predominant one, surrounded by Na^+^ ions.

## Introduction

Iodine is the heaviest stable halogen element. In spite of its low natural abundance, iodine is interesting in many research fields, ranging from Earth (*s.l*.) to materials sciences to waste management. In cosmochemistry studies, the ^129^I-^129^Xe decay is used as a geochronometer to date extra-terrestrial materials (*e.g*. meteorites)^1,2^ and to provide insights on the formation of planetary atmospheres^3–5^. Beyond geosciences, precursory iodine isotopes in the 131, 133 and 135 xenon decay chains are of particular interest for verification of the Comprehensive nuclear-Test-Ban Treaty (CTBT). Indeed, monitoring of the radioxenon isotopic radioactivity in the atmosphere is considered an efficient way of detecting and discriminating underground nuclear explosions after transport in the subsurface^6–8^. However, while Xe radionuclide source terms from an underground nuclear cavity can be calculated, still there are several uncertainties regarding iodine diffusion and mobilization, and iodine-magma interaction in nuclear cavities^9^. A major interest for iodine lays in the radioisotope ^129^I, which is present in many types of wastes, arising from the nuclear fuel cycle (NFC), as well as from research and medical applications. It is a long-lived fission product, with a very high solubility and mobility in the aqueous environment, either under oxidizing or reducing conditions, thus representing an environmental risk factor^10^. Despite its low concentration in nature, compared to the stable ^127^I, and the low energy of its beta particle^10^, ^129^I is also dangerous when entering the human body.

In nature, iodine occurs with different valence states, and respectively as iodide (I^−^), as the oxyanion iodate (IO_3_^−^) and rarely as elemental I (I_2_). Previous studies concerning iodine average valence and coordination environment were done in amorphous materials and cementitious wasteforms for nuclear waste management, or to determine I sorption on minerals *e.g*.^11–13^. It is quite important to evaluate the radionuclides oxidation state because besides the redox condition of the environment, also the speciation of the elements could influence their (re)distribution and transport in terrestrial materials and in the environment^10,14^. The immobilisation of radioisotope ^129^I via vitrification of wasteforms is considered inadequate due to the high volatility and low solubility of iodine in glasses^15^, even if relatively few data are available regarding iodine solubility in melts or in amorphous materials. Musselwhite and Drake^4^ incorporated up to 0.616 ± 0.013 wt.% I (~0.3 mol%) in synthetic glasses in the CaO-MgO-Al_2_O_3_-SiO_2_ system, and correlated iodine solubility with the NBO/T (non-bridging oxygens per tetrahedral cations) parameter, which is an old and simplistic concept^16^. Riley *et al*.^11^ studied iodine in low-activity borosilicate waste glasses. In this multicomponent system containing ~50 mol% SiO_2_ and ~9.5 mol% B_2_O_3_, the authors found iodine solubility to be around 1 wt.% (~0.5 mol%). McKeown *et al*.^15^ investigated as well multicomponent alumino-borosilicate glasses (SiO_2_/(B_2_O_3_ + SiO_2_) molar ratio ~0.8) with iodine contents ranging from 0.03 to 0.66 mol%. The authors reported that, in this system, iodine is mainly in its reduced form, and that in Na-bearing glasses, iodine is surrounded by an average of four Na^+^ ions with distances of ~3 Å, corroborating the previous results of Riley *et al*.^11^. Higher amounts of iodine can be incorporated in glass matrices as AgI; *e.g*. up to 28 mol% AgI in aluminophosphates^17^.

In order to identify the main parameters influencing iodine solubility, we investigated glasses in the Na_2_O-B_2_O_3_-SiO_2_ (NBS) system, where the SiO_2_/(B_2_O_3_ + SiO_2_) molar ratio was varied from 1 to 0. This system was selected because the thermodynamic properties and structure of NBS glasses are well-known^18^ and, furthermore, previous studies (ref.^19^ and references therein) indicated that the SiO_2_ content has a strong influence on the solubility of halogens. Two sets of experiments were carried out. The first one with the aim of establishing the solubility limit of iodine by adding different amounts of I_2_, and the second one to identify the variation occurring within the glass network upon ~1 mol% iodine incorporation.

## Experimental Methods

### Pristine glass synthesis

The investigated compositions are in the ternary system Na_2_O-B_2_O_3_-SiO_2_ (NBS). The borosilicate glasses have a constant amount of sodium (20 mol%) and a SiO_2_/(B_2_O_3_ + SiO_2_) molar ratio ranging from 1 to 0, *i.e*., from sodium-silicate to sodium-borate glass. The pristine glasses were made in a large batch from the appropriate amounts of oxide (SiO_2_), carbonate (Na_2_CO_3_) and acid (H_3_BO_3_). Before the mixture, the reagent powders were dried respectively, at 1000 °C, 350 °C and 100 °C and stored in a desiccator. After mixing the chemicals on stoichiometric proportions, the mixture was homogenized in an agate mortar, slowly heated in a platinum crucible to decompose the carbonate and borate components and melted between 950 and 1400 °C for a few hours to obtain bubble-free glass. The resulting melt was fast quenched by dipping the bottom of the crucible into water. To improve homogeneity of the glass, grinding and melting steps were repeated twice. No phase separation was observed, and all resulting glasses were transparent and clear. Chemical analysis of the pristine glasses shows that some Na losses occurred and the Na_2_O content within the sodium borosilicate glasses ranges between 13 and 20 mol%. Compositions and properties of the pristine glasses are reported in Table 1. The labels NBSx refer to sodium borosilicate glasses where *x* represents the SiO_2_ molar content.

**Table 1:** Analysed chemical composition (wt.%) and properties of the glasses.

Sample	T (°C)	P (bar)	Iodine source	starting amount I (mol%)	SiO_2_ (wt.%)	B_2_O_3_ (wt.%)	Na_2_O (wt.%)	I (wt.%)	Density (g/cm^3^)	Vm (cm^3^/mol)	Tg (K)	N_4_	SiO_2_/(SiO_2_ + B_2_O_3_)	notes
**pristine glasses**														
NBS80 air	1450				79.23	0	19.82	0	2.370 (9)	25.51			1.00	
NBS80	1400	1500			79.21	0	19.76	0	2.394 (2)	25.25	697.1		1.00	
NBS60 air	1100				58.35	23.2	19.48	0	2.495 (5)	25.02	855.2	0.71	0.74	
NBS60	1400	1500			57.48	22.87	19.47	0	2.512 (2)	24.85	854.7	0.74	0.74	
NBS40 air	1100				32.6	50.55	16.11	0	2.317 (12)	28.03	789.2	0.4	0.43	
NBS40	1400	1500			32.56	49.26	16.12	0	2.332 (1)	27.82	788.6	0.40	0.43	
NBS20 air	1100				14.39	72.17	14.59	0	2.176 (4)	30.75	740.2	0.32	0.19	
NBS20	1400	1500			14.59	66.92	14.49	0	2.198 (1)	30.38	718.4	0.28	0.20	
NBS00 air	950				0	87.86	12.62	0	2.078 (11)	32.99	699.2	0.21	0.00	
NBS00	1400	1500			0	82.93	11.74	0	2.075 (1)	32.99		0.23	0.00	White patina
**NaIO**_**3**_ **series: NBSx.y**														
NBS80.08	1500	1500	NaIO_3_	2	77.96	0	18.75	1.71 (8)	2.406 (3)	25.35	659.0		1.00	I_2_ bubbles inside the glass
NBS60.1	1500	1500	NaIO_3_	2	56.01	21.03	18.95	2.03 (9)	2.540 (2)	24.80	784.2		0.76	
NBS40.1	1500	1500	NaIO_3_	2	31.89	46.43	16.16	2.37 (6)	2.344 (1)	27.97	713.5		0.44	
NBS20.1	1500	1500	NaIO_3_	2	14.05	66.8	14.58	2.12 (6)	2.208 (1)	30.57	688.8		0.20	
NBS00.1	1500	1500	NaIO_3_	2	0.08	84.46	12.25	2.04 (5)	2.119 (3)	32.66	590.8		0.00	
**I**_**2**_ **series: NBSx-yI**_**2**_														
NBS80-1.07I_2_	1200	1500	I_2_	2	78.95	2.66	18.85	2.22 (12)	2.373 (1)	25.77	730.2		1.00	
NBS80-1.13I_2_	1200	1500	I_2_	10	76.62	2.11	18.48	2.28 (19)	2.316 (1)	26.42			1.00	I_2_ bubbles inside the glass
NBS80-0.8I_2_	1200	1500	I_2_	18.2	70.42	4.93	23.06	1.64 (70)	2.468 (10)	24.75			1.00	I_2_ bubbles inside the glass
NBS60-2.5*I_2_	1200	1500	I_2_	2.5	n.d.	n.d.	n.d.	n.d.						
NBS60-0.9I_2_	1200	1500	I_2_	9.5	59.51	22.74	14.98	1.88 (17)	2.495 (1)	25.24		0.68	0.75	heterogeneous
NBS60-0.8I_2_	1200	1500	I_2_	18.1	63.7	22.18	9.13	1.64 (23)	2.460 (1)	25.53			0.77	heterogeneous
NBS40-2.1I_2_	1200	1500	I_2_	18.5	33.64	51.07	6.56	3.91 (3)	2.135 (1)	31.17	536.2	0.17	0.43	white crystals
NBS20-1.9I_2_	1200	1500	I_2_	2	14.07	66.87	13.89	3.57 (5)	2.212 (1)	30.74	697.2	0.26	0.20	
NBS20-5.1I_2_	1200	1500	I_2_	10.1	13.85	63.35	10.61	8.99 (50)	2.189 (1)	31.99	590.0	0.20	0.20	Yellowish, Transparent
NBS20-4.3I_2_	1200	1500	I_2_	18	14.61	60.69	6.54	7.09 (13)	2.080 (7)	33.50	513.0		0.22	Orange, Transparent
NBS00-2.3I_2_	1200	1500	I_2_	2	0.07	82.33	11.25	4.17 (6)	2.100 (7)	33.32	499.0	0.22	0.00	
NBS00-9*I_2_	1200	1500	I_2_	9	n.d.	n.d.	n.d.	n.d.	2.177 (3)			0.18		

### HIP syntheses and iodine incorporation

Powdered pristine glass and iodine chemicals (I_2_ or NaIO_3_) were mixed by hand in an agate mortar. Batch of 300 mg of the powdered mixtures were inserted in platinum capsules and welded to avoid iodine leakage during experiments. Syntheses were carried out in a hot isostatic press (HIP) at Institut de Physique du Globe de Paris, as described in^14^. The HIP is a medium-pressure (up to 2000 bars) high-temperature (up to 2000 °C) device. The furnace is designed in graphite, and the pressure is provided by compressed gaseous Ar. The chamber is a large isothermal region, with less than 5 °C difference between two thermocouples adjacent to the samples. Variations between individual thermocouples indicate that run temperatures are uncertain by 20 °C and pressures are precise to ±5 bar, as assessed by^20^. Here we present data from experiments performed at medium pressure, where both pristine glasses and I-bearing ones were inserted together in the HIP, in order to obtain glasses prepared in the same pressure conditions. Pt capsules were cooled down at high pressure by switching off the heating elements, and decompression occurred at room temperature.

In this study, we report data for two glass series:

1. In order to assess iodine solubility limits, a full set of glasses has been doped with different amounts of crystalline I_2_ (from 1 up to ~18 mol%), and synthetized at 1200 °C and 1500 bar, for 20 h. These glasses are labelled NBSx-yI_2_, with x being the SiO_2_ and y the I molar contents (mol%).
2. In order to ascertain the variation occurring within the glass network upon incorporation of comparable amount of iodine (~1 mol% I), glasses were doped with NaIO_3_ as starting salt_,_ and synthetized at 1500 °C and 1500 bar for 7 h. Glasses within this second series are labelled NBSx.y, where x and y represent, respectively, the SiO_2_ amount and the measured iodine content (as mol%). Sample list and compositions of I-bearing glasses are reported in Table 1.

### Crystalline compounds

Several crystalline compounds were used in this study. All minerals are from the Mineralogy collection of Sorbonne University (Paris, F): iodargyrite (AgI), lautarite (Ca(IO_3_)_2_), salesite (CuIO_3_(OH)), and bellingerite (Cu_3_(IO_3_)_6_·2H_2_O). Other crystalline compounds investigated are: KIO_3_, NaIO_3_, NaI and KI. The eight crystalline compounds, representative of different structural environments around iodine (oxidation state, coordination, distances), have been investigated by Raman spectroscopy. The Raman spectra obtained have been compared with those available in the RRUFF database (http://rruff.info).

### Analytical techniques

Chemical compositions of all glasses have been analysed with a Cameca SX100 electron microprobe at Sorbonne University (Table 1). Boron nitride and copper iodide were used, respectively, as boron and iodine standards^14^. Ten measurements per sample (at 15 keV and 40 nA) were performed to obtain more representative values of boron and iodine contents; the average compositions are reported in Table 1. Boron calibration was achieved with BN as a standard and boron’s Kα absorption coefficients obtained by adjusting the other elements on analyses of several borosilicate glasses with variable B_2_O_3_ content from 80 to 0 mol%.

Raman spectra were recorded at room temperature using a T64000 Jobin-Yvon® Triple-spectrometer set up with a confocal system, and a 1024 CCD detector cooled by liquid nitrogen. A Coherent® laser 70 C Argon, with a wavelength of 488.1 nm, was used as excitation source providing 100 mW on the sample. All spectra were recorded between 16 and 1700 cm^−1^ with an integration time of 300 s. Linear baseline subtraction and normalization to the total area were performed with LabSpec® software.

Density measurements (Table 1) were done at room temperature by Archimedean method, using toluene as the immersion fluid.

Glass transition temperatures (Tg) were determined by Differential Thermal Analysis (DTA) with a Setaram 96 Line Evo apparatus. A blank analysis was performed before sample analysis to correct data from background signal. Temperature was increased with a rate of 5 °C/min and Tg was quantified by tangential method.

^11^B NMR spectra were acquired using a 20.0 T Bruker Avance III spectrometer (CEMHTI-CNRS, Orléans), operating at a Larmor frequency of 272.7 MHz. The samples were packed in 3 mm diameter AlN rotors and spun at 20 kHz using a Doty Scientific probe having an almost undetectable ^11^B background signal. We acquired the spectra using a single pulse sequence performed with a 20 kHz radio-frequency field and a 1.0 µs pulse length. Relaxation times were estimated using saturation-recovery experiment and found to decrease from approx. 6 s in pristine samples to approx. 2 s in loaded ones, with little difference between BO_4_ and BO_3_ components. We hence choose a 0.3 s recycle delay which, combined with the use of a small pulse angle, ensures quantitative response of both BO_4_ and BO_3_ components. The ratio N_4_ ([BO_4_]/([BO_3_] + [BO_4_])) is obtained by direct integration of each component on the experimental spectrum (Table 1), clearly separated on these experiments thanks to the use of a high magnetic field. MQMAS experiments have been acquired using a 2.5 mmm Bruker probe with samples spinning at 30 kHz. The triple-quantum z-filtered experiments were performed with excitation pulses applied at an RF-field of 125 kHz, leading to optimum pulses of 4.5 µs and 1.4 µs while the selective T_90_ pulse was 8.0 µs at an RF-field of 15 kHz. The indirect dimension was set to the spinning speed (i.e. 30 kHz) and 170 t_1_ increments were acquired using a 2 s recycle delay.

Iodine L_III_-edge X-ray Absorption Near Edge Structure (XANES) spectra were collected at the soft-XAS LUCIA beamline (SOLEIL, France) by using a Si_111_ crystal monochromator. The layout of LUCIA beamline is described in^21^. Iodine XANES spectra at the L_III_-edge (4557 eV) were collected in fluorescence mode (SDD), with the sample surface placed at 6 to 9 degrees with respect to the incoming beam, in order to avoid self-absorption. Some of the spectra were also recorded in Total Electron Yield (TEY) detection mode to verify self-absorption. The energy range scanned was from 4520 to 4680 eV with the absorption edge scanned with a step energy of 0.1 eV. An average of three spectra was taken, and the energy was calibrated by using a Ti foil (4966 eV). Five crystalline compounds (KI, NaI, I_2_, NaIO_3_, KIO_3_) were analysed as powder spattered on carbon tape. Glass samples were both analysed as bulk or powder.

## Results

### Glass properties and iodine incorporation

**Figure 1:**
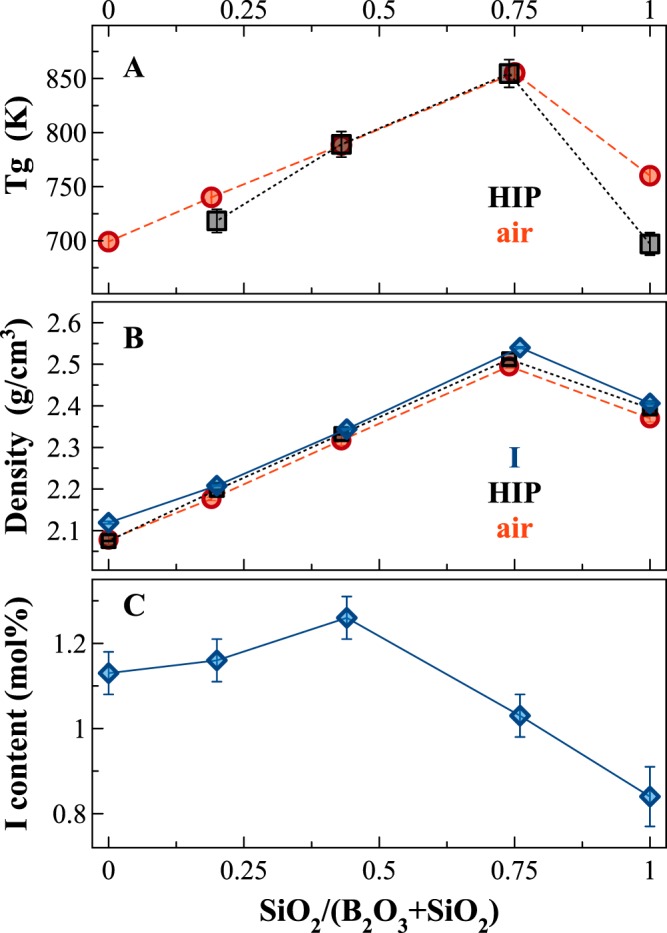
(**A,B**) Variations of glass transition temperature (Tg, K) and volumetric mass density (g/cm^3^) depending on the SiO_2_/(B_2_O_3_ + SiO_2_) molar ratio. (**C**) Amount of iodine incorporated within the glass network for the glass series NBSx.y (1500 °C and 1500 bar) vs. the SiO_2_/(B_2_O_3_ + SiO_2_) molar ratio. Errors within the points. Lines are guide for the eyes.

Figure 1A,B show the evolution of physical and thermal properties of the pristine glasses, synthetized in air or under pressure (HIP), depending on the SiO_2_/(B_2_O_3_ + SiO_2_) molar ratio. Both glass transition temperature (Tg) and volumetric mass density (density for short) increase smoothly until a SiO_2_/(B_2_O_3_ + SiO_2_) molar ratio = 0.75, and then sharply decrease. The substitution of Si for B induces differences in Tg and density up to 20–22%, in agreement with previous data in the NBS system^14,18^. The density of the I-bearing glasses is reported for comparison (Fig. 1B) and the data follow exactly the same trend as the pristine glasses, even if iodine samples are slightly denser than I-free glasses, with the B-end member being 2% denser.

The iodine content of the NBS glasses synthetized at 1500 °C and 1500 bar are reported in Fig. 1C. Depending on the SiO_2_/(B_2_O_3_ + SiO_2_) molar ratio, the amount of iodine incorporated varies from 0.84(7) to 1.26(5) mol% with a maximum for the glass having a SiO_2_/(B_2_O_3_ + SiO_2_) ratio ~0.5. B_2_O_3_-rich glasses have always higher amounts of iodine, compared to the SiO_2_-rich ones (Fig. 1C).

**Figure 2:**
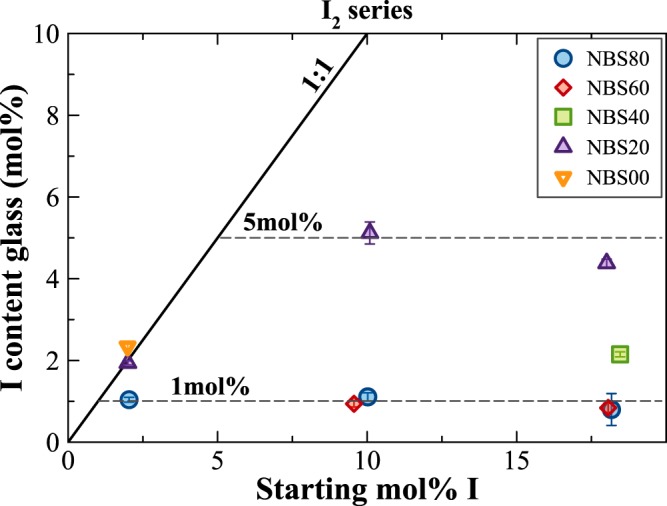
Measured iodine content in the glasses as a function of the starting amount of iodine, added as solid I_2_.

The second set of experiments (I_2_ series) was carried out to infer the iodine solubility limit in glasses, by adding different amounts of solid I_2_ in the Pt capsules. In Fig. 2, the nominal (starting) amount of iodine added is reported for the different glass compositions against the I content measured in the final glasses. Regardless of the starting amount, B_2_O_3_-rich glasses have always higher amounts of iodine compared to the SiO_2_-rich ones. Furthermore, silica-rich glasses were constantly partially crystallised and/or heterogeneous for doping level >5 mol%. The composition able to incorporate the highest amount of iodine is the NBS20, which has 5.12(27) mol% I. This value represents also the solubility limit for the borosilicate glass composition with 60 mol% B_2_O_3_ and 20 mol% SiO_2_ since higher doping levels did not produce any increase of iodine in the glass (Fig. 2). Silica-rich glasses have iodine contents up to ~1 mol% I.

### Structure of I crystalline compounds and I-bearing glasses

**Figure 3:**
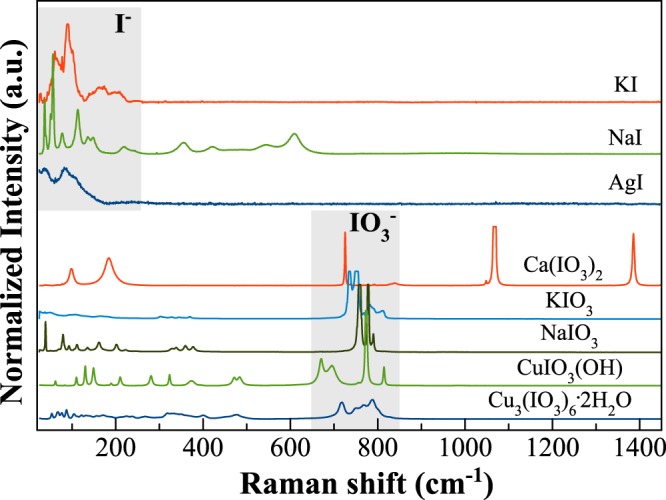
Raman spectra of iodine crystalline compounds. Shaded areas indicate the fingerprint area of iodide and iodate vibrations.

Raman signals for eight crystalline compounds having iodine in different structural environments (*i.e*. oxidation state, coordination, distances to first neighbours FN) are shown in Fig. 3. The acquired signals allowed to identify the different vibration modes attributable to iodine in the different environments. To avoid any alteration of the samples by the laser beam, spectra were acquired with a low power laser (~1 mW). Figure 3 clearly shows two main domains:

- Lattice modes “I-FN”: iodide compounds show the strongest vibrations in the frequency region 25–250 cm^−1^ ^22,23^, with the maxima peaking at ~56, 85 and 90 cm^−1^, respectively for NaI, AgI (iodargyrite), and KI. The electronic shells of halogen ions and the lattice vibrations of halogen and first neighbours (FN) play an important role in the Raman scattering, as can be seen from the spectra of the three iodide crystalline compounds reported in Fig. 3.
- IO_3_^−^ stretching region: iodate crystalline compounds have the strongest vibrations in the region 650–850 cm^−1^, with the exception of lautarite (Ca(IO_3_)_2_), which also has two strong bands at higher frequencies. KIO_3_ and NaIO_3_ have an orthorhombic structure where each I atom has three nearest O atoms, forming an IO_3_ group that provides several strong vibration modes above 200 cm^−1^, both related to IO_3_ group and internal vibration modes^24^. The iodate analysed here are crystalline KIO_3_ and NaIO_3_ (~752 and 758 cm^−1^, respectively), salesite (CuIO_3_(OH), at ~774 cm^−1^) and bellingerite (Cu_3_(IO_3_)_6_·2H_2_O, at ~788 cm^−1^).

In all crystalline compounds, independently of I valence, the nature of the atoms in the first and second coordination shells, along with their average distances, influence the vibrational frequency. Nevertheless, we identified two characteristic regions for iodide and iodate compounds, respectively centred around 100 cm^−1^ and 750 cm^−1^ (shaded areas in Fig. 3).

In order to assess structural variations in the glass network, first of all, a detailed analysis of the pristine glasses prepared in air or under pressure was carried out by Raman and NMR spectroscopy (see Supplementary Materials). Afterward, to evaluate the influence of iodine incorporation on the glass structure, we performed Raman and ^11^B NMR analysis on B- and I-rich glasses of the first series, namely NBS20 compositions doped with crystalline I_2_.

Raman spectra in the frequency range 20–1700 cm^−1^ contain the vibrational contributions related to B a/o Si. Tri-fold and four-fold coordinated boron units show vibrations in the range between ~1200 and 1600 cm^−1^, whereas the vibrational modes of SiO_4_ tetrahedra are in the 850–1250 cm^−1^ frequency region. Bands in the 350–1150 cm^−1^ region can be related to vibrations of the Si tetrahedra-rings (~400–650 cm^−1^) and to vibrations of BO_3_ and BO_4_ units located in different structural groups (*e.g*. boroxol rings, triborates, pentaborates, danburite). The asymmetric stretching modes of silicon in the high frequency portion are labelled according to Si polymerization: *Q*^*0–4*^, where *Q* represents the Si centred tetrahedron, and *0–4* represents the number of bridging oxygens (BO).

**Figure 4:**
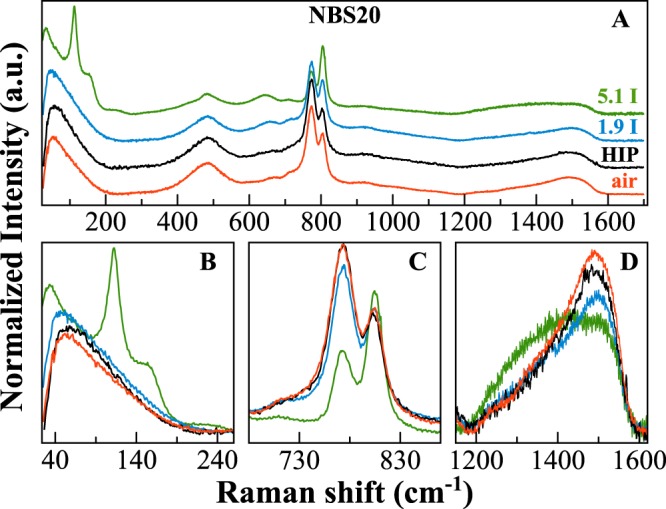
Raman spectra of glasses with composition NBS20, I-free done in air or under pressure (HIP) or doped with ~1.9 and ~5 mol% of I_2_ (blue and green lines, respectively for NBS20-1.9I_2_ and NBS20-5.1I_2_).

Raman spectra of iodine-bearing NBS20 glasses are shown in Fig. 4 along with those of the pristine glasses. All bands are very broad and characteristic of borosilicate glasses^18^. The main differences between undoped and doped glasses are observed in the very low frequency region (<250 cm^−1^; Fig. 4B), in the intermediated region (600–900 cm^−1^; Fig. 4C), and in the *B*-range (1200–1600 cm^−1^; Fig. 4D). The Boson peak^25^ in amorphous B_2_O_3_ has been associated to out-of-plane librations of boroxol rings and BO_3_ triangles^26^. This feature shifts from 58 cm^−1^ in the HIP pristine glass to 46 and 34 cm^−1^ in iodine-bearing glasses (respectively for NBS20–1.9I_2_, NBS20-5.1I_2_). Moreover, in the very low frequency region three other broad contributions appear in the I rich glass, at ~112, 160 and 235 cm^−1^. Those three contributions are not visible in the NBS20-1.9I_2_ glass, even if there is a slight increase of the background and a shift toward lower frequencies (Fig. 4B). In the intermediate frequency region, the presence of iodine causes a decrease of the bands related to pentaborate rings (770 and 732 cm^−1^), and a clear increase of the boroxol band (805 cm^−1^). In the *B*-range, the band at higher frequency (~1500 cm^−1^) strongly decreases its intensity for I-bearing glasses (Fig. 4D).

**Figure 5:**
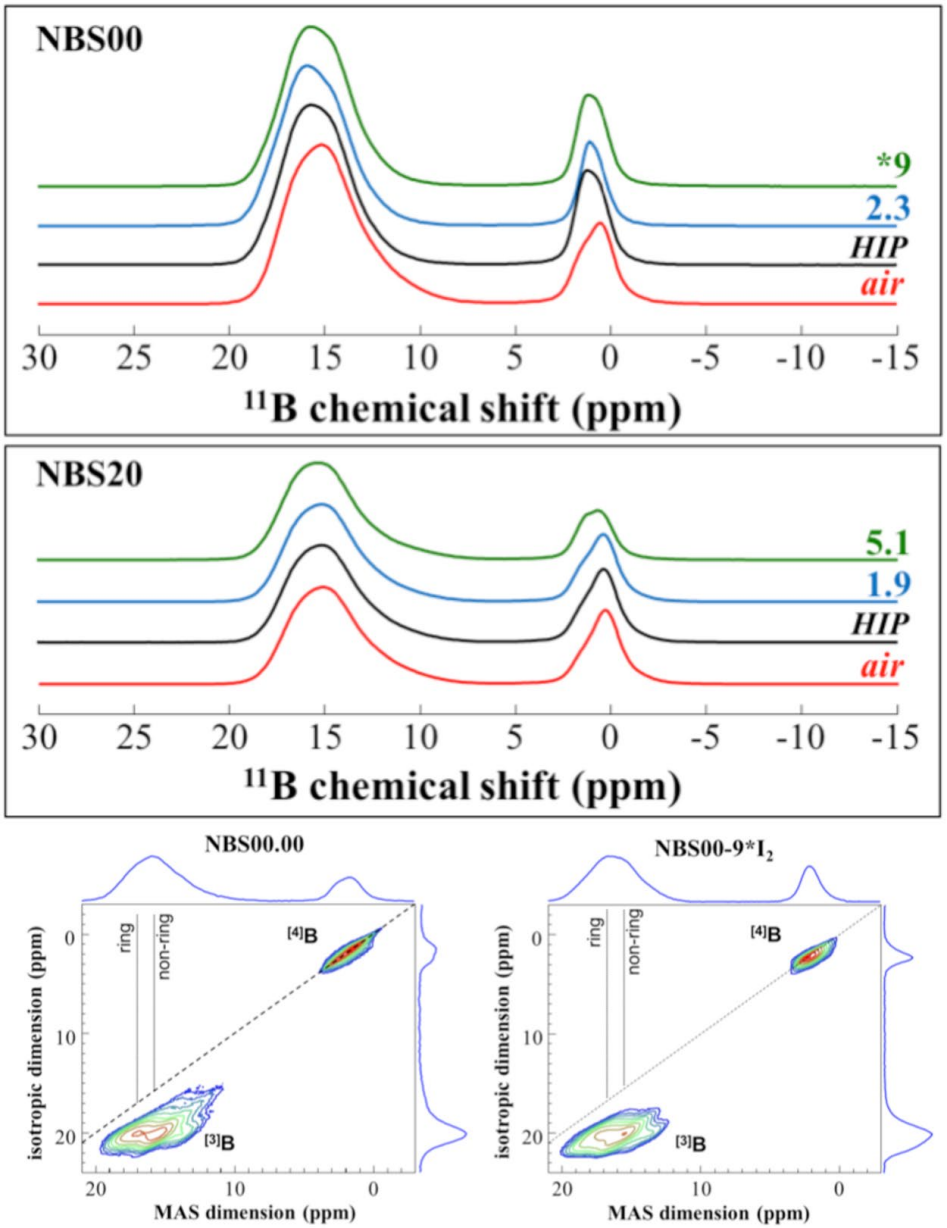
(**A**) ^11^B MAS-NMR analysis done at 20 T for pristine glasses synthetized in air, under pressure (HIP), and with different amounts of iodine (mol%). (**B**) ^11^B 3QMAS analysis done at 20 T for the boron end-member pristine glasses synthetized in air and doped with a nominal amount of 9 mol% I_2_ (NBS00-9*I_2_).

^11^B MAS NMR spectra have been collected for borosilicate glasses doped with different amount of iodine, added as crystalline I_2_ (Fig. 5A). ^11^B MAS NMR allows to quantify the structural variations occurring in the B environment depending on the SiO_2_/(B_2_O_3_ + SiO_2_) molar ratio and I presence. Signals from ^[3]^B ([BO_3_]) and ^[4]^B ([BO_4_]) units centred respectively, around 15–16 ppm and 0 ppm, are very well resolved. The former is associated to tri-coordinate boron [BO_3_], either ring type in boroxol units or involving both BO_3_ and BO_4_ ([BO_3_] ring) or non-ring type^27,28^, whereas the contribution around 0 ppm is associated to tetra-coordinated boron [BO_4_]. The narrow tetrahedral boron peak band is the results of different overlapping contributions, with a band related to boron surrounded by four silicon atoms (usually around −2 ppm), and to boron surrounded by one boron atom and three silicon atoms (^[4]^B:1B, 3Si) at ∼0 ppm^27,28^.

The incorporation of high amounts of iodine induces visible structural variations in the [BO_3_] and [BO_4_] units. Indeed, adding iodine in B-rich compositions decreases the [BO_4_] components and increases the contribution around 16 ppm corresponding to the [BO_3_] units (boroxol rings). Sample NBS20-5.1I_2_ has the highest amount of incorporated iodine (~5 mol%) and it presents the strongest modifications in the B environment, with a net decrease of the ^[4]^B:1B, 3Si component at ∼0 ppm in the NMR spectrum. To obtain more detailed structural information, ^11^B 3QMAS spectra were collected for the B end-member glass synthetized in air, and the one doped with a nominal amount of 9 mol% I (Fig. 5B). As seen from the isotropic dimension spectra (displayed vertically in Fig. 5B), the variations in the [BO_3_] ring/non-ring distribution and the decrease of the [BO_4_] are confirmed.

### Iodine L_III_-edge X-Ray Absorption Spectroscopy

**Figure 6:**
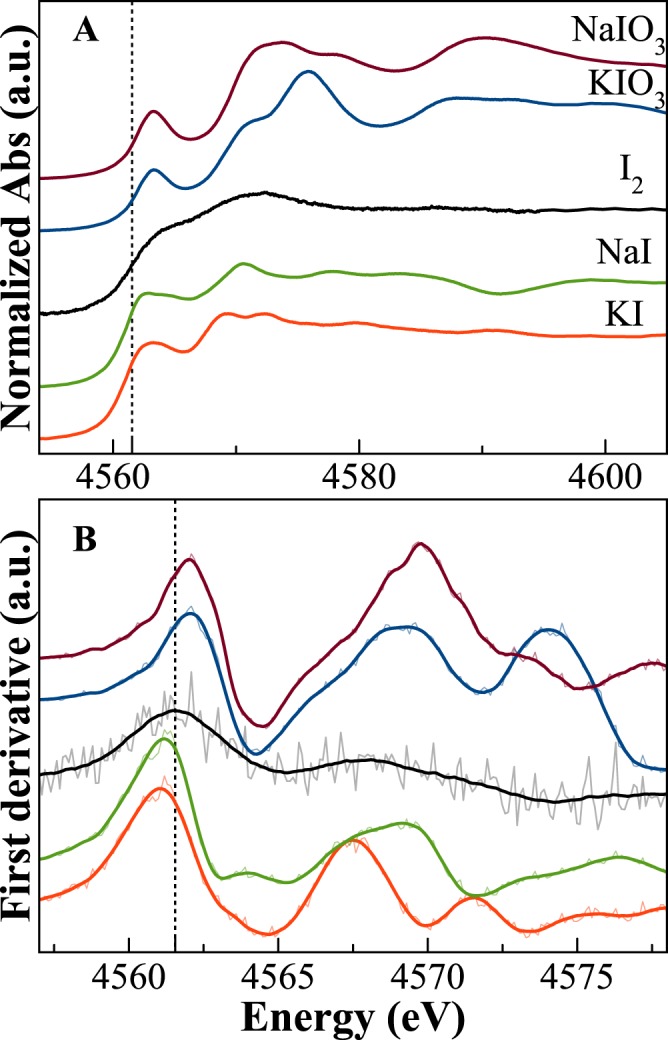
Normalised iodine L_III_-edge XANES spectra of iodine crystalline compounds (**A**), and first derivative (**B**) of the spectra. The black vertical dotted line indicates the position of the edge for crystalline I_2_. Edges for iodate compounds are at higher energies, whereas iodides have their absorption edges negatively shifted by ~0.5 eV.

Figure 6 shows the I L_III_-edge spectra for the five model compounds analysed, along with their first derivatives. All compounds have relatively broad bands associated with *2p*-> final *d-*state transitions. The spectra of crystalline iodine compounds show several differences depending on iodine oxidation state and local surrounding, with a clear shift of the absorption edge toward higher energies by increasing the valence. The absorption edge for crystalline I_2_ is at 4561.55 eV (dotted vertical line in Fig. 6) and the spectrum shows only another evident contribution at higher energy (~6.2 eV). Iodide model compounds have their absorption edges negatively shifted of about ~0.5 eV and show clear post-edge structures, whereas iodate compounds have the absorption edges positively shifted of ~7.6 and 8.0 eV (for KIO_3_ and NaIO_3_, respectively) and show strong features, both above and below the absorption edge. The changes observed in the post edge features of iodide and iodate compounds are mainly related to the different iodine local environments, both in terms of coordination and bond distances.

**Figure 7:**
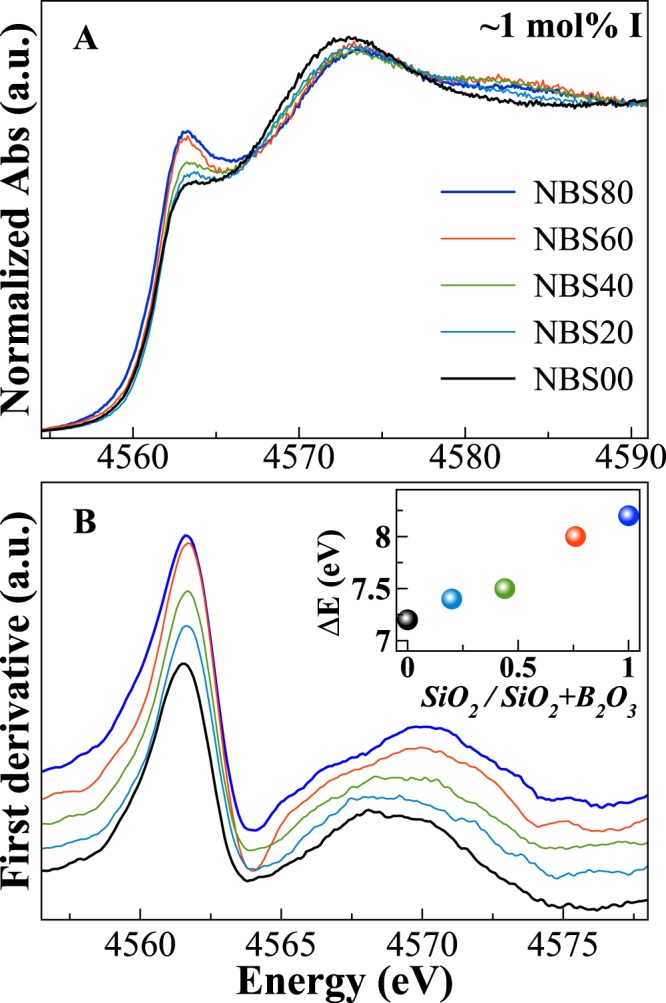
Normalised I L_III_-edge XANES spectra of iodine bearing glasses in the NBSx.y series (**A**) and first derivative of the spectra (**B**). The energy splitting between the shoulder and the main peak is reported in the inset (errors within the symbols).

Iodine L_III_-edge XANES spectra have been collected for glasses with different SiO_2_/(B_2_O_3_ + SiO_2_) molar ratios and similar iodine contents (NaIO_3_ series: NBSx.y, Fig. 7A). I-bearing glasses have broader peaks compared to the crystalline compounds and only three main features can be observed. A shoulder around ~4561.8 eV, the main edge ~7–8 eV positively shifted, and a broad contribution at ~4580.8 eV (all energies refer to the position of the maxima of the first derivative, Fig. 7B). Based on the energy positions for the different glasses compared to the model compounds, it seems that iodine is stabilized in all glasses with mixed valences, and the average I redox state is thus intermediated between +5 and −1.

Changes in the SiO_2_/(B_2_O_3_ + SiO_2_) molar ratio go with several changes in all three main features, with the borate-end member (NBS00.1) having the most smoothed spectrum. The energy splitting between the shoulder and the main peak systematically increases by increasing the SiO_2_ content (inset in Fig. 7B).

## Discussion

### Iodine solubility

The SiO_2_/(B_2_O_3_ + SiO_2_) molar ratio strongly influences iodine incorporation and the main cation building the glass network affects glass homogeneity. Indeed, I-bearing samples in B_2_O_3_-rich glasses are always transparent, glassy and homogeneous, whereas the SiO_2_-rich glasses are partially crystallized or heterogeneous.

B_2_O_3_-rich glasses always incorporate higher amounts of iodine compared to SiO_2_-rich ones and two solubility limits have been identified: ~5 mol% I as a limit for the B_2_O_3_-rich composition (SiO_2_/(B_2_O_3_ + SiO_2_) = 0.2), and a solubility limit of ~1 mol% I was established for the two SiO_2_-rich glasses (Fig. 2).

The higher glass homogeneity and iodine solubility in B_2_O_3_-rich borosilicate glasses could be related to the glass structure. SiO_2_-rich glasses, NBS80 and NBS60, are based on cations mostly 4-fold coordinated (SiO_4_ and BO_4_ units). By substituting B for Si, the proportion of BO_4_ sharply decreases and, as a result, all properties (density, Tg, viscosity) decrease as well. The fragility of a glass^29^ reflects to what degree the temperature dependence of the viscosity deviates from the linear behaviour. For example, in a pure SiO_2_ glass, this dependence can be well-represented as an Arrhenian behaviour, and the liquid is called “strong”. NBS80 (Si end-member) approximately exhibits linearity and as reported by^18^ glasses become more fragile by increasing B content. Here, we relate glass fragility (and in turn, the viscosity dependence) to iodine incorporation. Hence, we contemplate that the stronger the glass network, the lower the iodine content.

**Figure 8:**
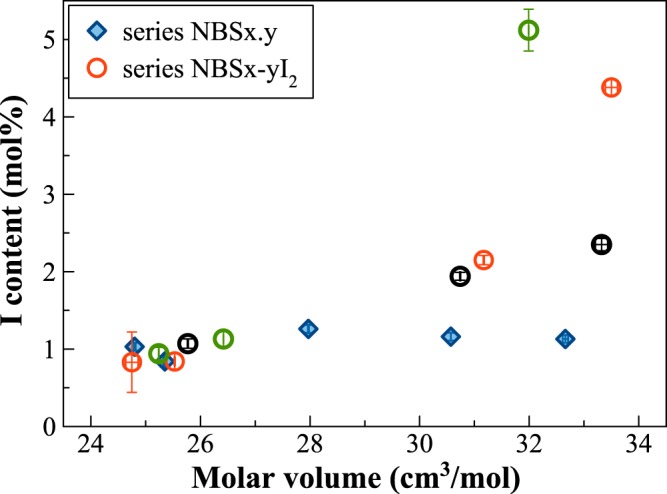
Iodine content against molar volume of the glasses: diamonds, glasses in the NBSx.y series. Glasses in the NBSx-yI_2_ series are represented by various colours depending on the amount of iodine added to the pristine glass (*i.e*. black circles: 2 mol%; red circles: 5 mol%; green circles: 10 mol%). All values are reported in Table 1.

Musselwhite and Drake^4^ observed an increasing iodine solubility with higher concentrations of network-forming species for glasses in the CaO-MgO-Al_2_O_3_-SiO_2_ (CMAS) systems, and thus by increasing the glass polymerization. They used the NBO/T parameter (non-bridging oxygens per tetrahedral cations) to describe this dependence. However, it must be considered that this parameter is too simplistic to represent a glass network, because it implies assumptions on cation speciation. The authors considered indeed, Al to be fully 4-fold coordinated, and Ca to act only as a network modifier, even if the Al_2_O_3_/CaO molar ratios varied from 2.2 to 0.8 across the studied CMAS glasses. Thus, the observed dependence may have been over-interpreted. Musselwhite and Drake^4^ also reported a well-defined positive linear correlation between iodine solubility and molar volume of the glasses, similarly to the behaviour observed for noble gases^30,31^. The molar volume values for the I glasses are reported in Fig. 8, both in the NBSx.y and NBSx-yI_2_ series. Glasses in the NBSx.y series, independently from the SiO_2_/(B_2_O_3_ + SiO_2_) molar ratio, have similar iodine contents (0.84–1.26 mol%) with a maximum for the glass with a SiO_2_/(B_2_O_3_ + SiO_2_) ratio of 0.44. Thus, the correlation between molar volume and iodine content is not verified (NBSx.y glasses in Fig. 8). On the other hand, glasses in the NBSx-yI_2_ series show an increase of iodine content with the molar volume of the glass, with the B-rich glasses having at least twice the amount of incorporated iodine compared to the Si end-member (Fig. 8). The molar volume could therefore be another parameter to get some insights on the network adaptation and, thus, to predict the available space in the glass structure. However, it is important to point out that the cation valence might influence the molar volume calculations.

### Iodine effect on glass properties and structure

In our study, we carried out both element specific techniques to probe the local environment around boron and iodine and Raman spectroscopy to provide an overview of the structural changes upon B/Si substitution and iodine incorporation.

Boron environment in glasses is a combination of both tri- and tetra-coordinated sites, the relative amount of each depending on the SiO_2_/(B_2_O_3_ + SiO_2_) molar ratio, and on iodine amount. The variations observed in the macroscopic properties (Tg, density, Fig. 1) are strictly linked to the fractions of [BO_3_] and [BO_4_] units. For example, NMR data of sample NBS60 (see Fig. S1) shows the highest amount of 4-fold coordinated B (N4 = ~0.7), in agreement with the presence of the danburite-like ring signal observed in the Raman spectra^32^. The relatively higher amount of ^[4]^B tetrahedral units involves the alkali cations to act as charge balancers to stabilize the 4-fold coordinated boron, thus the formation of non-bridging oxygens (NBO) is hampered. Consequently, the glassy network is more polymerized and Tg increases (Fig. 1A).

**Figure 9:**
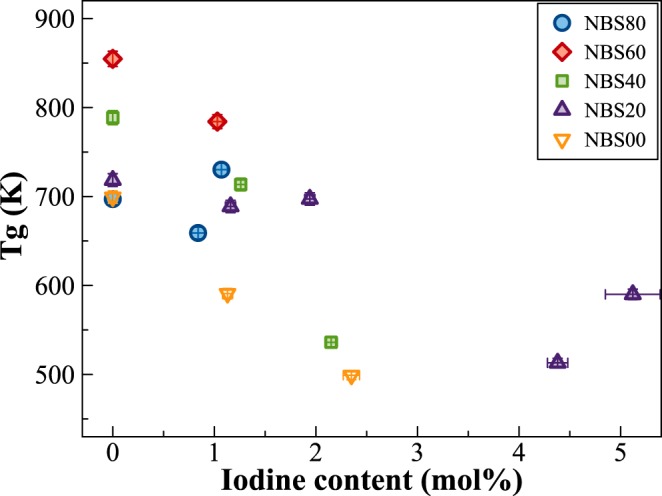
Variation of the Tg values depending on the iodine content for all glasses here investigated, both in the NBSx.y and in the NBSx-yI_2_ series. Values from Table 1.

Iodine incorporation induces higher amount of [BO_3_] units compared to [BO_4_], especially [BO_3_]-ring. Usually, in alkali borate glasses, the stabilization of [BO_4_] units is due to an electron from the alkali cation used by the boron to form the fourth B-O bond. Hence, local charge neutrality is provided by the positive alkali ion adjacent to the negative BO_4_^−^ unit^33^. In iodine-bearing glasses, being the alkali content constant, the decrease of the tetra-coordinated B could consequently be explained by considering Na^+^ ions surrounding the I^−^ species, in agreement with the observation done by Raman spectroscopy (Fig. 4). Indeed, the Raman broad peaks in the very low frequency region (at ~112, 160 and 235 cm^−1^) well-match vibrations of NaI and can be assigned to the vibrations of iodine ions I^−^ next to Na^+^ ions. This assumption could also explain the variations observed at the macroscopic scale. With iodine incorporation triggering a loss of sodium ions in the Si-B network, and in turn, an increase of [BO_3_] units, the network polymerization is expected to decrease. A decrease of the glass transition temperature when iodine enters into the glass network is observed indeed (Fig. 9). Even considering the scatter of some of the data, the general trend indicates that the higher the iodine content, the lower the glass transition temperature, whatever the composition.

### Iodine speciation

Following the approach of Schlegel *et al*.^34^, in order to evaluate the variations observed in the iodine XAS spectra, we do consider the literature data available on bromine [[*Ar*] *3d*^10^ *4s*^2^ *4p*^5^], since it has properties (e.g. electronegativity, electron affinity, ionic radius) intermediate between Cl and I [[*Kr*] *4d*^*10*^ *5s*^*2*^ 5*p*^5^]. Matsuo *et al*.^35^ studied Br compounds at the L_III_ and L_II_-edges, and calculated the partial density of states which indicates that the electronic transitions responsible for the XANES L-edges features are mainly due to transitions from Br *2p* to the unoccupied *4d* electronic level, which is split in two sublevels. Thus, depending on the photoabsorber symmetry, the ligand field splits the unoccupied orbitals into sublevels at higher and lower energies (*e*_*g*_ and *t*_*2g*_, respectively). The difference in energy (ΔE) between these two sublevels is related to the ligand field potential which increases by decreasing the average distance between absorbing element and the first coordination shell. Considering reasonable that similar electronic transitions are related as well to the iodine L_III_-edge features, we evaluated the energy difference between the first two peaks. As observed in Fig. 7, the energy splitting ΔE systematically increases from borate to borosilicate to silicate glasses, suggesting that the average distances between iodine and first neighbours decrease by substituting Si for B. The different iodine speciation in Si-rich and B-rich glasses is also emphasized by the broad contribution at ~4581 eV, which is clear in Si-rich glasses, decreases in intensity for NBS20 and disappears in the B end-member. Modulations of the contributions close to the main absorption edge are mainly related to the different iodine local environments, both in terms of coordination number and bond lengths, thus confirming that iodine speciation is different in B-rich glasses compared to the Si-rich ones.

Previous XAS studies on iodine in borosilicate glasses (I K-edge)^11,15^ suggested I^−^ as the main species dissolved in the glasses, and an iodide-like environment. This means ~ four Na^+^ ions in the first coordination shell, with shorter average <I-Na> bond distances compared to the NaI crystalline phase. In our glasses, the XANES spectra only display two to three main features and the broadening of the peaks confirms the disordered nature of the I surrounding. The edge energy positions are intermediate between iodide and iodate compounds suggesting that different I species coexist in the glass matrix, albeit with higher proportion of reduced species. We can assume the partial decomposition, at high temperature, of the starting iodate salt IO_3_^−^ to I_2_ and I^−^, with an equilibrium established between these species in the molten material:

$$I{O}_{3}^{-}+6{e}^{-}\leftrightarrow 5{I}^{-}+3{O}^{2-}$$

Depending on the viscosity of the melt, (i) the diffusion of the different species will be strongly affected, and (ii) there will be different proportions of dissolved I^−^ and I^5+^ ions in the glass matrix and gaseous I_2_ eventually trapped in bubbles. Since the viscosity of B-rich glasses is much lower than the viscosity of Si-rich ones, and because the split between the XANES features is decreasing with increasing B content, we could assume that a higher amount of I^−^ species are dissolved in B-rich glasses. Hence, an iodide-like environment is proposed, with Na^+^ ions surrounding the I^−^ species. On the contrary, for Si-rich glasses, the lower average distances between absorbing elements and the first coordination shell is the result of the higher melt viscosities that hamper the distribution and diffusion of I species into the glasses. This also explains the lower I solubility, and the higher heterogeneity for Si-rich glasses.

**Figure 10:**
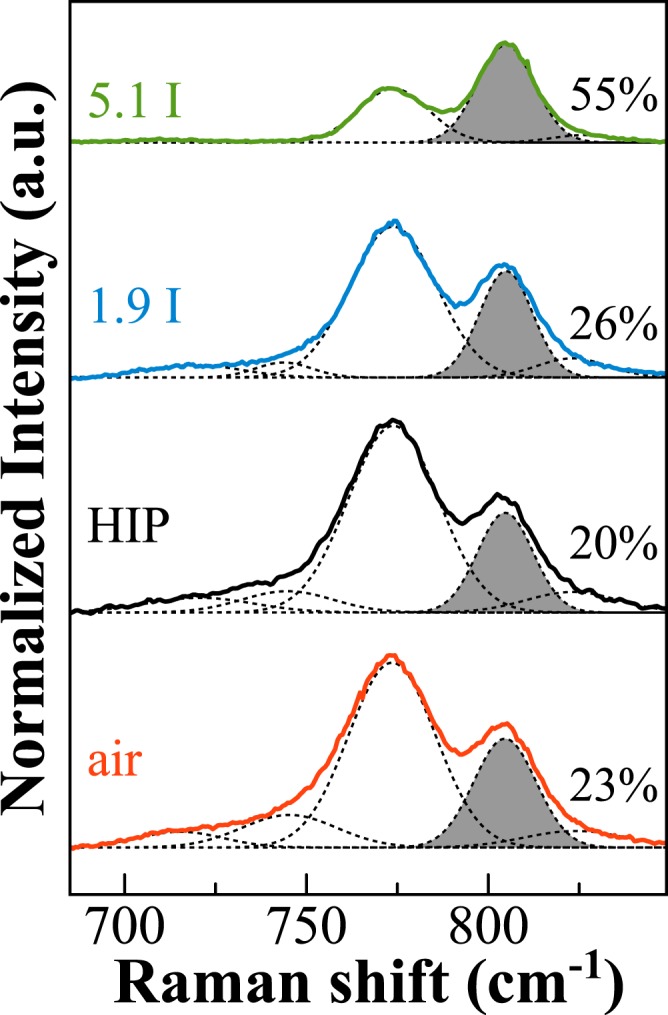
Deconvolution of the Raman frequency region 650–900 cm^−1^ for the NBS20 pristine and NBS20-yI_2_ glasses. The boroxol component at ~805 cm^−1^ is highlighted and the relative area of this component, with respect to the total area, is reported.

The coexistence of different I species within the glasses and the occurrence of <Na-I> environments corroborates the observations done both by NMR and Raman spectroscopy. As previously explained, the three broad contributions in the Raman spectrum of the I rich glass are compatible with lattice mode vibrations of iodide species, and in particular with those of NaI. Moreover, NMR data indicate that there is a strong decrease of the [BO_4_] component and an increase of [BO_3_]-ring units, confirming that Na^+^ ions are not anymore acting as charge balancer of [BO_4_] units. Therefore, all these analyses point to the main presence of Na^+^ ions next to iodide species. On the other hand, the increase of the [BO_3_]-ring component by NMR does not match the strong increase of the Raman signal related to the boroxol-rings. The deconvolution of the 650–900 cm^−1^ Raman region indicates that the relative area of the peak at 805 cm^−1^ for the I-bearing glass is twice higher than for the I-free samples (Fig. 10). Thus, it is reasonable to assume that a further vibration causes the strong increases of the peak, with a frequency that is compatible with the IO_3_^−^ stretching modes, in agreement with the XAS data, that indicate the coexistence of reduced and oxidized I species.

## Conclusions

This study focuses on the incorporation of iodine in glasses in the ternary system Na_2_O-B_2_O_3_-SiO_2_. The SiO_2_/(B_2_O_3_ + SiO_2_) molar ratio strongly influences iodine incorporation. In silicate glasses (Si-rich), the solubility of iodine is much lower than in borate and B-rich glasses. This composition dependence has been explained by considering the network adaptation (the available space in the structure) and the fragility parameter of the glass network, and we suggest that the stronger the glass network, the lower the iodine content.

Furthermore, our results suggest the stabilization of iodine with different valences within the glasses. Raman spectroscopy of I-bearing glasses reveals the presence of two characteristic contributions around 100 cm^−1^ for iodide and around 750 cm^−1^ for iodate, and XAS data confirm the coexistence of iodine with different valences, with the main species being the reduced one. The incorporation of iodine in glasses triggers variations in the network connectivity, because iodine dissolves mainly as I^−^ which is surrounded by Na^+^ ions. Hence, iodine does not play directly the role of network-modifier in the glass but contributes to change the structure by affecting the structural role of the network-modifier/charge balancer elements (*i.e*. sodium or potassium)^14^.

Network modifier cations play a major role in influencing the solubility of iodine. The higher viscosities of Si-rich glasses, compared to the B-rich ones, limit I diffusion and dissolution. Moreover, in silicates, the alkali ions create NBO and induce depolymerisation, which implies that only a few Na ions will be available to surround iodine, and to form Na-I complexes. Thus, the solubility of iodine is limited for high silica content. Borate and B-rich glasses can create a stable network based only on [BO_3_] species, that together with the higher network adaptability, make Na^+^ ions available to form Na^+^-I^−^ and Na^+^-IO_3_^−^ complexes, enhancing iodine solubility.

## Supplementary information

Supplementary Materials
