# Supplementary material for: Iodine solubility and speciation in glasses
Source: Sci Rep. 2019 May 23;9:7758. doi: 10.1038/s41598-019-44274-4 (PMC6533311; doi:10.1038/s41598-019-44274-4)
Supplement: Supplementary file 1 — Supplementary Materials [file 41598_2019_44274_MOESM1_ESM.pdf]

Iodine solubility and speciation in glasses.

M.R. Cicconi[1], E. Pili[2], L. Grousset[1], P. Florian[3], J.C Bouillard[4], D. Vantelon[5], D.R. Neuville[1]

[1] Institut de Physique du Globe de Paris, Équipe Géomatériaux, CNRS-UMR7154, Sorbonne Paris Cité, 1 rue Jussieu 75005 Paris cedex 05, France

[2] CEA, DAM, DIF, F-91297 Arpajon, France

[3] CNRS, CEMHTI UPR3079, Université d'Orléans, F-45071 Orléans, France

[4] IMPMC-Sorbonne Université, 4 place Jussieu 75252 Paris cedex 05, France

[5] SOLEIL Synchrotron, L'orme des merisiers, Saint Aubin BP48, 91192 Gif-sur-Yvette Cedex, France

SUPPLEMENTARY MATERIALS

Structure of pristine borosilicate glasses

In order to assess variations in the glass network upon iodine introduction, the detailed analysis of the pristine glasses, either prepared in air, or under pressure was carried out by Raman and NMR spectroscopy.

^{11}B MAS NMR spectra (Figure 1S) allowed to quantify the structural variations occurring in the B environment depending on the $SiO_2/(B_2O_3+SiO_2)$ molar ratio. Signals centred around 15-16 ppm and 0 ppm, are very well resolved. The former is associated to tri-coordinate boron $[BO_3]$ ($^{[3]}B$), either ring type in boroxol units or involving both $[BO_3]$ and $[BO_4]$ units ($[BO_3]$-ring) or non-ring type, outside the rings ($[BO_3]$-non-ring) [1,2]. The contribution around 0 ppm is associated to tetra-coordinated boron $[BO_4]$, and this narrow tetrahedral boron band results from different contribution overlapping, with a band related to boron surrounded by four silicon atoms (usually around −2 ppm) and boron atom surrounded by three silicon atoms and one boron atom ($[BO_4]$:1B, 3Si) at ~ 0 ppm. [1,2].

Sample NBS60, having almost equal amount of B_2O_3 and Na_2O (*R* ratio = $[Na_2O/ B_2O_3]$ ~ 0.95) has the highest amount of $[BO_4]$ species, since trigonal $[BO_3]$ units are converted to $[BO_4]$ tetrahedra. By substituting boron for silicon, the component at ~ -2 ppm decreases, thus the relative amount of ($[BO_4]$:1B, 3Si) units decreases, and the amount of trigonal $[BO_3]$, especially in the ring units, increases (Figure 1S). At the same time a peak around 2 ppm is growing with decreasing amount of SiO_2, evidencing the formation of a borate sub-network where $[BO_4]$ units are linked mostly to $[BO_3]$ but also possibly to $[BO_4]$ ones [3]. The integrated area below the curves, respectively for the contributions of $[BO_3]$ and $[BO_4]$ was calculated and the $[BO_4]/([BO_3]+[BO_4]$ ratio (N_4) was quantified for all glasses analysed (see Table 1).

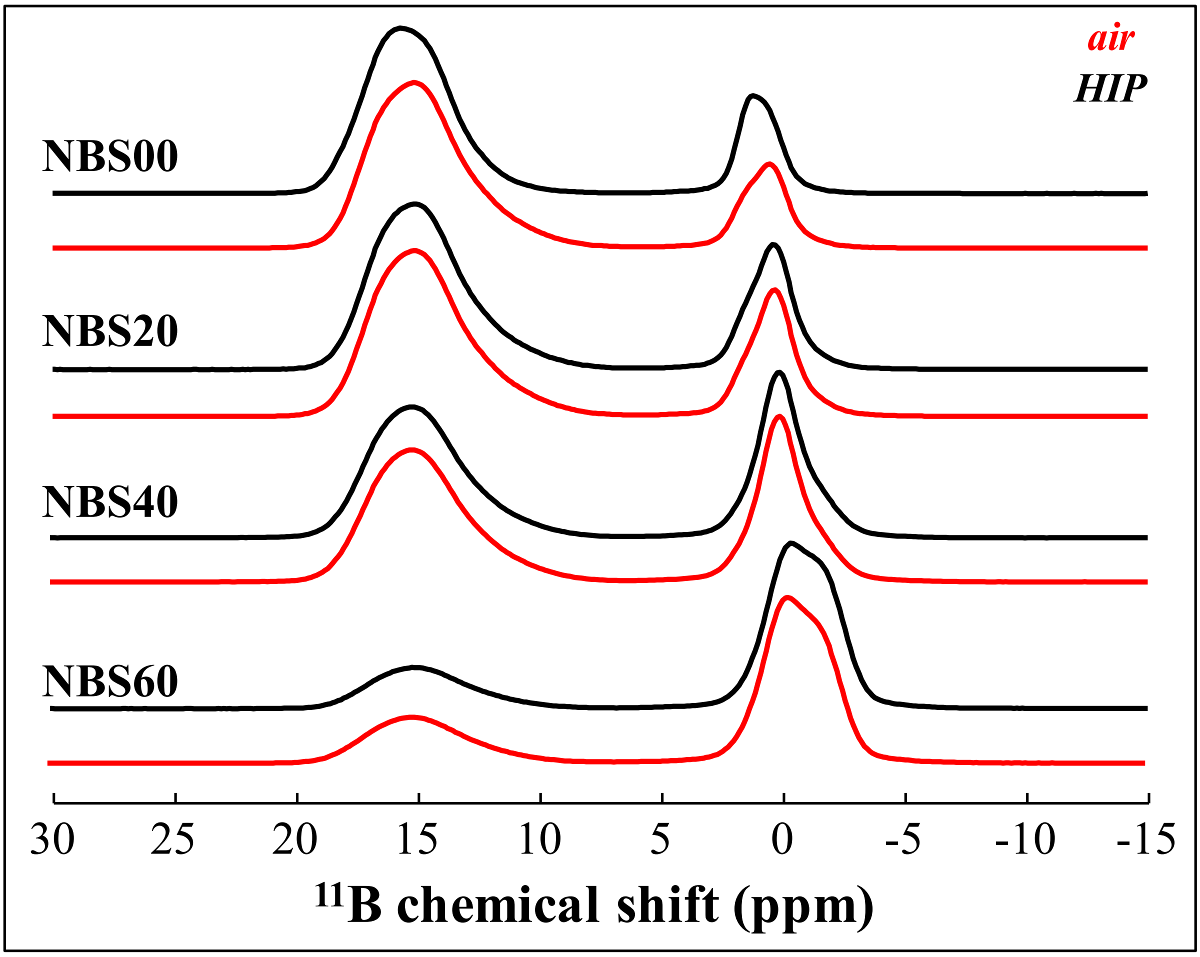

Figure 1S – ^{11}B MAS-NMR analysis done at 20T on pristine glasses prepared in air or under pressure (HIP).

Raman spectra, in the frequency range 20-1700 cm^{-1} (Figure 2S), show the vibrational contributions related to B a/o Si. Tri-fold and four-fold coordinated boron have vibrations in the range between ~ 1200 and 1600 cm^{-1}, whereas the vibrational modes of SiO_4 tetrahedra are in the 850-1250 cm^{-1} frequency region. Bands between 350-1150 cm^{-1} can be related to vibrations of the Si tetrahedra-rings (~ 400-650 cm^{-1}) and vibrations of BO_3 and BO_4 units located in different structural groups (*e.g.* boroxol rings, triborates, pentaborates, danburite). The asymmetric stretching modes of silicon in the high frequency portion (Q_{Si} range: 850-1250 cm^{-1}) are labelled according to Si polymerization: Q^{0-4}, where Q represents the Si centred tetrahedron, and *0-4* represents the number of bridging oxygens (BO).

Figure 2S shows the normalised Raman spectra of pristine glasses produced in air, or under a pressure of 1500 bar (HIP). The B end-member glass (NBS00) done under pressure, had a white patina, and the few sharp peaks in the high frequency region of the Raman signal

confirmed a partial crystallization of the sample. All other glasses were transparent and homogeneous and the Raman spectra show their amorphous nature [4–7].

The Si end-member Raman spectrum fully resembles the signal of a Na-tetrasilicate glass [8], with an intense contribution at high frequency (Q_{Si}-range centred at ~1105 cm^{-1}), which arises from the different symmetric stretching motions of silica tetrahedra. In the lower frequency portion (300-700 cm^{-1}) the main peak at ~526 cm^{-1} is associated to vibration modes of Si tetrahedra-rings, whereas the small shoulder at 600 cm^{-1} is attributed to breathing modes of 3-membered rings (D_2). By substituting B for Si, there is a net decrease of the Q_{Si}-range intensity and the rise of two contributions: the first one at ~630 cm^{-1}, attributed to breathing modes of borosilicate danburite-type rings, and the second one is a high frequency broad band (1200-1600 cm^{-1}) related to tri-fold and four-fold coordinated boron (*B*-range). With increasing B content (NBS40 and NBS20), the SiO_2 vibration modes almost disappear, whilst the B-related band in the high frequency region strongly increases and become more asymmetric. Two relatively sharper peaks rise at ~ 770 cm^{-1} and 805 cm^{-1}, respectively assigned to borate and boroxol rings (Figure 2S) [4–7].

Under a moderate applied pressure (HIP samples), Si-rich glasses (NBS80 and NBS60) show a decrease of the splitting between the low frequency main band and the Q_{Si}-range. The centroid position of the Q_{Si}-range for the Si end-member glass done under pressure is shifted of circa -5.3 cm^{-1}, and the deconvolution of this portion indicates that the relative area of Q^4 species decreases (-12%). Moreover, there is an intensity decrease of the D_2 line. This decrease of polymerization agrees with the lower Tg value of glass NBS80 HIP.

Glass NBS60 done under pressure has the low frequency main band shifted toward higher frequencies (~ +3.4 cm^{-1}) while the *B*-range centroid position is shifted toward lower frequencies (~ -5.5 cm^{-1}) because of the rise of the component around 1325 cm^{-1}. Sample NBS40.0 shows no significant changes, whereas the B-rich borosilicate glass (NBS20) shows strong changes both in the high and low frequency portions. The total area of the *B*-range is 11% lower for sample NBS20 HIP because of the decrease of the BO_3 components, providing indeed a [BO_3]/([BO_4] ratio ~9.6% lower. The main peaks in the NBS20 Raman spectra are relatively sharp and synthesis under pressure induce a decrease of the boroxol component (~805 cm^{-1}) with respect to the pentaborate rings (~770 cm^{-1}) (Figure 2S).

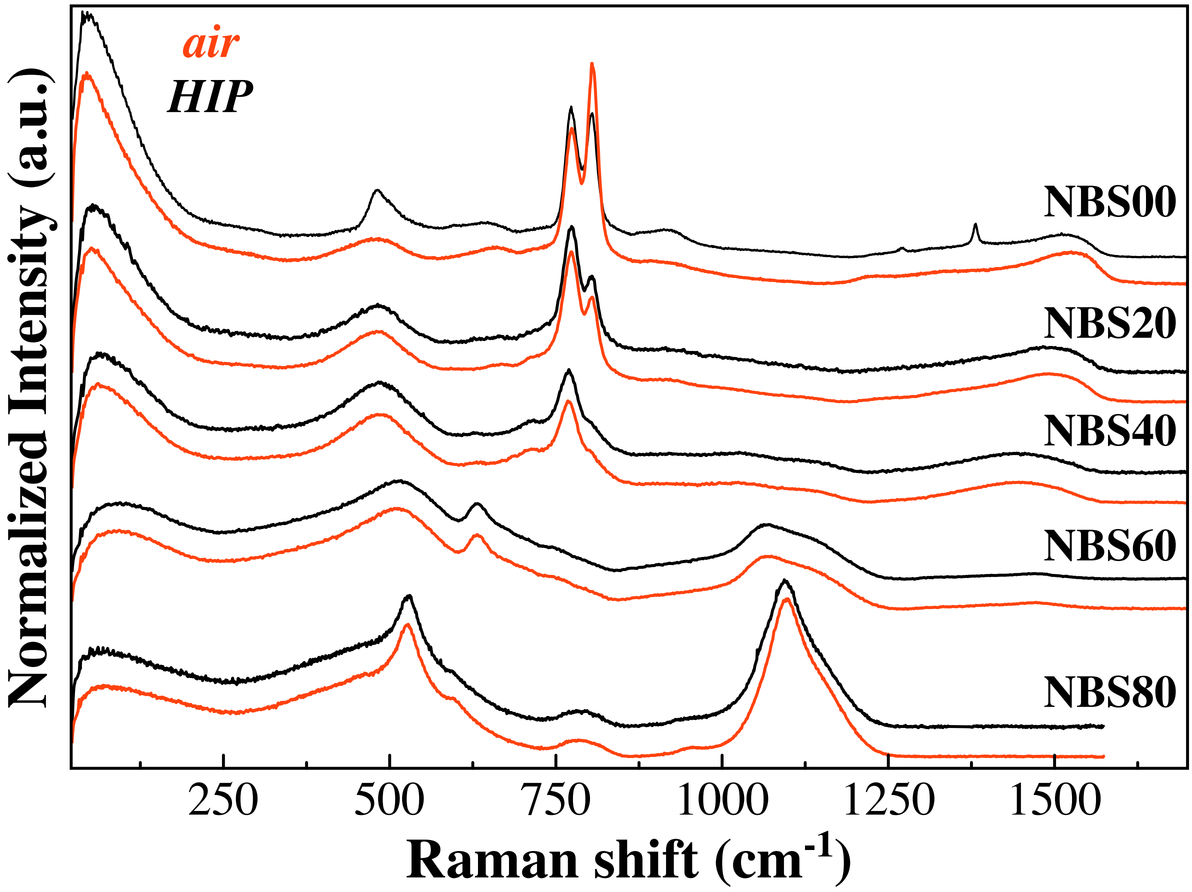

Figure 2S – **Raman spectra for pristine glasses pristine glasses done in air or under pressure (HIP).**

Variations in the structure of pristine glasses synthetized in air, or under pressure (HIP) were observed both by Raman and NMR spectroscopy (Figures 1S and 2S), for the borate glass (NBS00) and for the B-rich borosilicate glass (NBS20). Especially in the latter, despite the clear differences observed, it is mandatory to contemplate that both synthesis temperature and quenching rate strongly influence the structure of borosilicate glasses [2], thus we cannot ascribe the changes observed exclusively to the effect of pressure. On the other hand, we must consider that I-bearing glasses and I-free HIP glasses were prepared with similar experimental conditions, thus limiting any synthesis bias or thermal history effect. Therefore, for I-bearing glasses, it is reasonable to ascribe all structural changes mostly to iodine incorporation within the glass network.

REFERENCES

1. Du, L.-S. & Stebbins, J. F. Solid-state NMR study of metastable immiscibility in alkali borosilicate glasses. *J. Non. Cryst. Solids* **315,** 239–255 (2003).
2. Angeli, F. *et al.* Effect of temperature and thermal history on borosilicate glass structure. *Phys. Rev. B* **85,** 054110 (2012).
3. Möncke, D., Tricot, G., Winterstein-Beckmann, A., Wondraczek, L. & Kamitsos, E. I. On the connectivity of borate tetrahedra in borate and borosilicate glasses. *Phys. Chem. Glas. J. Glas. Sci. Technol. Part B* **56,** 203–211 (2015).
4. Bunker, B. C. Multinuclear nuclear magnetic resonance and Raman investigation of sodium borosilicate glass structures. *Phys. Chem. Glas.* **31,** 30–41 (1990).
5. Bunker, B. C., Kirkpatrick, R. J., Brow, R. K., Turner, G. L. & Nelson, C. Local Structure of Alkaline-Earth Boroaluminate Crystals and Glasses: II, 11B and 27Al MAS NMR Spectroscopy of Alkaline-Earth Boroaluminate Glasses. *J. Am. Ceram. Soc.* **74,** 1430–1438 (1991).
6. Yano, T., Kunimine, N., Shibata, S. & Yamane, M. Structural investigation of sodium borate glasses and melts by Raman spectroscopy.: I. Quantitative evaluation of structural units. *J. Non. Cryst. Solids* **321,** 137–146 (2003).
7. Yano, T., Kunimine, N., Shibata, S. & Yamane, M. Structural investigation of sodium borate glasses and melts by Raman spectroscopy. II. Conversion between BO4 and BO2O− units at high temperature. *J. Non. Cryst. Solids* **321,** 147–156 (2003).
8. Lenoir, M., Grandjean, A., Linard, Y., Cochain, B. & Neuville, D. R. The influence of Si,B substitution and of the nature of network-modifying cations on the properties and structure of borosilicate glasses and melts. *Chem. Geol.* **256,** 316–325 (2008).
